# When Interference Helps: Increasing Executive Load to Facilitate Deception Detection in the Concealed Information Test

**DOI:** 10.3389/fpsyg.2013.00146

**Published:** 2013-03-28

**Authors:** George Visu-Petra, Mihai Varga, Mircea Miclea, Laura Visu-Petra

**Affiliations:** ^1^Applied Cognitive Psychology Center, Department of Psychology, Babes-Bolyai UniversityCluj-Napoca, Romania; ^2^Developmental Psychology Lab, Department of Psychology, Babes-Bolyai UniversityCluj-Napoca, Romania; ^3^COGNITROM LtdCluj-Napoca, Romania

**Keywords:** deception detection, concealed information test, interference design, executive functions, cognitive load

## Abstract

The possibility to enhance the detection efficiency of the Concealed Information Test (CIT) by increasing executive load was investigated, using an interference design. After learning and executing a mock crime scenario, subjects underwent three deception detection tests: an RT-based CIT, an RT-based CIT plus a concurrent memory task (CITMem), and an RT-based CIT plus a concurrent set-shifting task (CITShift). The concealed information effect, consisting in increased RT and lower response accuracy for probe items compared to irrelevant items, was evidenced across all three conditions. The group analyses indicated a larger difference between RTs to probe and irrelevant items in the dual-task conditions, but this difference was not translated in a significantly increased detection efficiency at an individual level. Signal detection parameters based on the comparison with a simulated innocent group showed accurate discrimination for all conditions. Overall response accuracy on the CITMem was highest and the difference between response accuracy to probes and irrelevants was smallest in this condition. Accuracy on the concurrent tasks (Mem and Shift) was high, and responses on these tasks were significantly influenced by CIT stimulus type (probes vs. irrelevants). The findings are interpreted in relation to the cognitive load/dual-task interference literature, generating important insights for research on the involvement of executive functions in deceptive behavior.

## Introduction

There is a growing body of behavioral, psychophysiological, and neuroimaging evidence revealing that lying is a complex, cognitively demanding behavior. Most of this evidence reflects an overall increase in executive control demands imposed by lying, as compared to truth-telling. Truth-telling is considered a baseline, almost automatic cognitive state (Spence, 2004). To support this claim, lying has been proven to take longer than truth-telling (Spence et al., 2001), necessitating greater cognitive effort (see Vrij et al., 2011, for a recent review). Furthermore, it activates a wider network of prefrontal neural areas linked to executive functioning (see Christ et al., 2009; Gamer, 2011, for reviews). However, recent research questions the “cognitive complexity” view of deception (Gombos, 2006), revealing that in certain contexts lying might not be that cognitively demanding, especially as a result of extensive practice (e.g., Hu et al., 2012a,b; Van Bockstaele et al., 2012). This raises the need for developing deception detection tools less vulnerable to the effects of practice. One interesting possibility is to increase the cognitive workload experienced during deceptive behavior (Vrij et al., 2006). Inducing an overall increase in cognitive/executive load, such as by asking participants to narrate their deceptive stories backwards has been shown to interfere with lying, facilitating the process of lie detection by enhancing verbal and non-verbal cues to deception (Vrij et al., 2008). However, the backwards recall technique has been questioned with regard to the accuracy and completeness of the retrieved information (Dando et al., 2011), suggesting that a global interference with deceptive and memory processes might induce some unwanted collateral effects. An ingenious recent study (Debey et al., 2012) actively manipulated executive control, using an ego depletion procedure *prior* to detecting deception and inducing goal neglect *during* the deception task (by using longer response-stimulus intervals). Across two experiments, goal neglect, but not the ego depletion procedure facilitated deception detection efficiency, generating longer deceptive response speed (but not consistently lower accuracy).

Vrij et al. (2006) suggested that requiring interviewees to perform a *concurrent* secondary task while being interviewed might provide a useful tool to enhance lie detection. There have been some preliminary experimental attempts to add a parallel task aimed at disrupting the executive functions involved in the deceptive act, yielding mixed evidence in terms of effects on deception. In a Concealed Information Test (CIT, see the description below), Ambach et al. (2008) added a parallel inhibition (Go/No-Go) task. This manipulation was supposed to interfere with the very sub-processes of response inhibition that are required for deceptive responses. However, the physiological and behavioral measures of deception (RTs, error rates) were not significantly affected by introducing this additional measure (see Ambach et al., 2008 for a discussion of these negative findings). In a recent investigation, Ambach et al. (2011) pursued this line of reasoning, but they introduced a working memory (WM) task in parallel with the deception test. This manipulation affected RTs to critical items to a larger extent when compared to irrelevants. Considering the limitations induced by the very long RTs specific to the psychophysiological measurement design, the authors suggested that a faster pace of the task (asking the subjects to respond within a second) would enhance this preliminary documented effect. This idea was recently tested by introducing an interfering inhibition (dot-probe) task within each trial of the Reaction Time-based (RT-based) CIT, which led to an increase in its detection efficiency (Hu et al., 2013). The present study aimed at further testing this prediction, using the RT-based CIT at a faster pace, and interfering with two different executive functions shown to be involved in the deceptive act (WM updating and shifting). Moreover, rather than introducing a parallel task, peripheral to the deception detection task, in the present study the concurrent task targeted the same items used in the deception detection test, presumably creating a larger interference with deceptive behavior.

The abovementioned “high cognitive workload” studies have used a variety of deception detection paradigms, ranging from naturalistic interviewing settings to elaborated experimental contexts. The use of a unique and well-supported research paradigm which has also been used in ecological settings would substantially benefit the integration of various investigations targeting cognitive control in the deceptive act. This research context could be provided by the CIT, which is one of the most widely adopted techniques by nowadays deception research (Verschuere et al., 2011; Ben-Shakhar, 2012). Originally known as the Guilty Knowledge Test (Lykken, 1959, 1974), this procedure is an interrogation technique designed to test individuals for knowledge that only a guilty person could posses. The subject is presented with several multi-choice questions. For each question, there are several equally plausible alternatives, only one being correct. Hence, the test is based on the rationale that the critical alternative is recognized only by the guilty suspects. A different version of this test based on measuring reaction times was proposed by Seymour et al. (2000), now known as the RT-based CIT (Seymour and Kerlin, 2008; Verschuere et al., 2010). In this procedure, the subject is required to give speeded responses to three types of items: probes, targets, and irrelevants. Probe items are selected from the crime itself and are supposed to represent relevant details of the crime; the irrelevant items share a variable degree of categorical similarity with the relevant items, and are usually several times more numerous. The deceptive subject denies recognition of both irrelevant and probe items. Target items (explicitly learned and recognized as such) are used in order to prevent the subject from entering an automatic mode of responding; they also share categorical similarity with the other two types of items. A number of studies have suggested that this procedure can successfully differentiate between truthful and deceptive responses, or between guilty and innocent participants on the basis of RTs, supporting the validity of the RT-based CIT (see Verschuere and De Houwer, 2011 for a recent review).

The main aim of the present study was to systematically investigate whether introducing a concurrent executive load targeting the very CIT items, rather than a parallel interfering task, would better differentiate between truthful and deceptive responses in the RT-based CIT. The current investigation used an interference design, introducing tasks involving two executive functions evidenced to be relevant for the deceptive act: memory updating and flexible set-shifting (Morgan et al., 2009; Visu-Petra et al., 2012). In order to efficiently plan and execute a deceptive act, a person needs to continuously monitor and update memory contents in order to distinguish truthful from deceptive responses, and to flexibly alternate between these mental sets in producing the deceptive response (Walczyk et al., 2003). A third executive functioning dimension (according to the model proposed by Miyake et al., 2000), namely inhibition, has been documented to be involved in deception (Verschuere et al., 2007; Hu et al., 2013), but it was not directly targeted by the current study.

Consistent with previous findings by Ambach et al. (2011), we hypothesized an increase in CIT detection accuracy due to the introduction of the concurrent memory load condition. We anticipated that the introduction of the requirement to hold on to a memory load while performing recognition judgments would interfere with WM updating processes, and disrupt their efficiency by slowing them down (Logan, 1979). The manipulation was supposed to affect deceptive responses to a greater degree than truthful responses, because they required a larger amount of executive resources compared to simple visual recognition skills necessary for responses to irrelevants and targets, and the executive resources are depleted by the concurrent task. This would be evidenced by an increase in difference scores (RTs) between probes and irrelevants in the CIT plus memory condition. A second research interest was to investigate whether this effect could be replicated when introducing a concurrent task which required flexible set-shifting. We explored whether performance slowing would be further increased in this context, because flexibly shifting between responses in a trial-to-trial manner could place greater executive demands than a simple memory load. In addition to the main measure derived from the CIT (the RT), we wanted to explore whether response accuracy would discriminate between truthful and deceptive responses in the three experimental conditions. Finally, we wanted to see whether performance on the concurrent tasks itself would be more impaired on the trials containing probes than on the trials with irrelevants, thus reflecting the reciprocal interference generated by deception-related increased executive demands.

## Materials and Methods

### Participants

Participants (*N* = 75, 62 females) were recruited from general psychology classes by using an online recruitment system and received credit for their participation. All participants underwent the mock crime procedure described below, followed by the three CIT conditions. Data from the CIT plus memory test of one participant were lost due to a technical failure, and data from one participant were discarded altogether from the analysis because he remembered less than four of the five probes used in this experiment. A remaining total of 73 participants (62 females) were included in the data analyses. The age of participants ranged from 19 to 43 years, and the mean age was 22.76 years (SD = 4.79). Participants had normal or corrected-to-normal vision and wore glasses or contact lenses if necessary.

### Materials

Concealed information test items were two-word phrases: five probes, five targets, and 20 irrelevants (four corresponding to each probe), which were generated for this study and very similar to items used in previous studies (e.g., Farwell and Donchin, 1991; Seymour et al., 2000; see Appendix). They were displayed using the E-Prime software on a 17″ monitor. Each word pair subtended 0.85°of vertical visual angle, and ranged from 2″ to 4.2″ of horizontal visual angle depending on word pair length, from a viewing distance of approximately 60 cm.

### Tasks

All participants completed a series of tasks as follows: they read the instructions for the mock crime, they executed the mock crime, then completed a filler task; afterward, they studied and learned the target items and finally resolved the three CIT conditions (the order of presentation was counterbalanced across subjects).

#### Mock crime

The participants were initially required to read and sign the informed consent form. Afterward, the mock crime scenario was presented. Written instructions were used at this time, according to which they had to pretend to be a student of Psychology who was about to take a previously failed exam at an important course in the following day. Because of some personal issues, he/she had been unable to study. However, in the previous day, the student had presumably visited the professor’s office for a meeting. There he/she noticed a paper on the desk and saw the login Id (Psiho MCC, where the MCC abbreviation stands for – in Romanian – Cognitive Behavioral Modifications, the actual name of the course) and password (*patru verde/four green*) for the discipline’s e-mail account which is hosted on the faculty’s official web site. With this information, he/she was instructed to access the course e-mail account from a *café* (*Café Amber*) placed in certain *street* (*Bicaz Street*; all locations were chosen from another city in order to avoid previous exposure). After accessing the account (which was created to be identical to a real course application on the actual faculty website), the participant had to search the Inbox for the e-mail with the exam subjects that the professor had sent to the course *tutor* (*Amalia Ciuca*; the name of the actual tutor was used, with her and the professor’s consent) for multiplying exam papers. The participant had to forward this message with the attachment to their personal e-mail account.

Subjects read these written instructions twice and memorized (emphasized) the five critical items (i.e., the probes). Afterward, they were asked to go into a distant room of the same building (designated as Café Amber) and perform the actions from the scenario (access the e-mail account with the username and password, forward the e-mail). The interface was a mock program designed for this study and was deactivated after the completion of the study.

Following the mock crime, a non-verbal reasoning test taken from a standardized battery was used as a filler task, lasting for about 12–15 min. This data was not analyzed further.

In the target learning phase, the participants learned a sequence of five items similar to the probes. They were instructed to memorize the items in order to reproduce and recognize them. In order to obtain a good memory for the target items the participant was asked to complete two pencil-and-paper cued recall tests after the memorizing phase: in the first run, they were presented with the first word of the two-word phrase, and in the second run they were presented with the second word. In each run the participant completed the missing item. This was followed by a free recall test. If wrong answers were given at any time, they were again presented with the items and asked to memorize them. A final verbal recall was performed to ensure a good retention of each item.

#### RT-based CIT

After the mock crime and the target learning phase, the participants undertook the three CIT procedures designed for this study: a classical RT-based CIT, a CIT with a concurrent memory task (CITMem), and a CIT with a concurrent set-shifting task (CITShift).

The items utilized in this study were two-word phrases belonging to three categories of items: *probes* (the five critical items from the mock crime), *targets* (five to-be-recognized items, also from the same category as the probes), and *irrelevants* (items from the same category as the probes, not previously encountered). For each probe, four similar irrelevants were selected. The items were matched on number of syllables across the three categories (see Appendix). In each of the three conditions, each item was repeated four times, generating a total of 120 trials/condition. The participants were instructed to press Yes when presented with the targets, indicating recognition, and No to any other item encountered. The two response keys were counterbalanced across subjects. Item presentation was randomly established by the E-Prime software for the CIT and CITShift conditions. For the CITMem, a randomized list was generated and kept constant across subjects, to allow for verbal recall accuracy to be checked by the experimenter with a response key.

In the CITShift, the primary task remained the same, but the stimuli themselves appeared written in bold or in italics. Subjects had to press the answers to the CIT *once* if the item was written with *bold* and *twice* if the item was written with *italics*. Stimuli were presented equally often in bold or italics. The assignment of number of presses to the respective fonts was also counterbalanced across subjects.

In the CITMem condition, the task was spaced in sequences consisting in groups of three items, with items randomly divided over sequences. The subject again had to press Yes or No to each item according to CIT instructions, but additionally he/she had to memorize the last word of each two-word item. After each three items sequence, a blank screen appeared. The subject had to verbally reproduce the three words he/she had memorized. After this, the participant pressed the space bar in order to initiate the next three items sequence. The experimenter verified the accuracy of verbal answers with an answer-key. A total of 40 memory checks were performed.

Each condition began with a training phase identical in length (16 trials). For each condition, written instructions were presented and verbally clarified by the experimenter. The instructions for the CIT were identical for all the three tasks. For the CITMem and CITShift, general CIT instructions were followed by specific instructions referring to the additional task. A shortened version of the instructions also appeared on the computer screen before the practice trials. The items used in the training phase were similar to the subsequent CIT items (three probes, three targets, 10 irrelevants).

The inter-stimulus interval randomly varied between 500, 800, and 1100 ms in order to discourage automatic responses or preparation effects (cf. Seymour et al., 2000). If a response was not made within 1200 ms, a “Too slow” message appeared. The 1200 ms interval was established after a pilot study in which shorter stimulus presentation RTs were associated with floor levels of performance on the CITMem and CITShift. No feedback was given (except for the practice trials, where the participant received feedback after every response). Each item remained on the screen until a response was made.

#### Scoring

For each condition, accuracy and RT (for accurate responses) on the CIT according to stimulus type represented the main collected measures. On the CITMem, an additional index of memory for each stimulus type across trials, and also for mixed groups of three was added. For each group of three items, we checked whether they recalled the last word for irrelevants, probes, or target items, and whether the group of three items was also correctly recalled. For the CITShift, accuracy in pressing once/twice the answer according to stimulus font was calculated; however, an inaccurate shift was not considered to be an error on the CIT (e.g., if the subject pressed once the answer No when presented with a probe it was scored as a shifting error, if the task was to press twice, but it was not scored as a CIT error). However, in the analysis of RTs, only time until first press was recorded and analyzed (for correct CIT responses).

## Results

### Response time

#### Group effects

**Figure 1:**
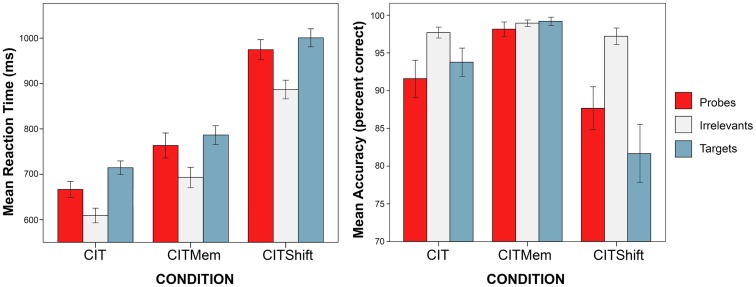
**Mean response time (*left*) and accuracy (*right*), according to stimulus type (Probe, Irrelevant, or Target) and condition (CIT, CITMem, CITShift)**. Error bars indicate standard error of the mean (±2 SEM).

In order to analyze the RT data, an elimination of outliers was first conducted. Since there was an established upper limit for RTs of 1200 ms, we only eliminated responses faster than 200 ms as outliers. Descriptive data for accuracy and response time according to stimulus type are presented in Figure 1. In the subsequent analyses only the comparison between probes and irrelevants is considered, similar to other studies using the RT-based CIT (Seymour et al., 2000; Seymour and Kerlin, 2008; Verschuere et al., 2010; Visu-Petra et al., 2012).

A two-way repeated-measures ANOVA with Condition (CIT vs. CITMem, and CITShift) and Stimulus type (probe vs. irrelevant) as within-subject factors was conducted for the mean RT data. The results showed that there was a significant effect of Condition, *F*(2, 144) = 341.91, *p* < 0.001, MSE = 9600.04, partial η^2^ = 0.83. *Post hoc* pairwise comparisons (with a Bonferroni correction) indicated that subjects were significantly faster on the traditional CIT than on both the CITMem, and the CITShift, *p* < 0.001. They were also significantly faster on the CITMem than on the CITShift.

There was a significant main effect of Stimulus type, *F*(1, 72) = 288.63, *p* < 0.001, MSE = 1958.06, partial η^2^ = 0.80. Across conditions, subjects were faster in responding to irrelevants than to probes, *p* < 0.001 (see Figure 1).

Finally, there was a significant Condition × Stimulus type interaction, *F*(2, 144) = 12.5, *p* < 0.001, MSE = 678.88, partial η^2^ = 0.15. There was a significant increase across tasks in RTs to both irrelevants, and probes, respectively, with the fastest responses on the CIT, followed by responses on the CITMem, and by longest responses on the CITShift, *p* < 0.001 in each case. To investigate the magnitude of the difference between RTs for irrelevants and probes across conditions, difference scores (difference between mean RTs for probes minus mean RTs for irrelevants) were calculated for each condition. *Post hoc* paired *t*-tests revealed that RT differences were smaller in the CIT than in the CITMem, *t*(72) = 2.12, *p* = 0.04, and in the CITShift, *t*(72) = 5.23, *p* < 0.001. Additionally, difference scores were significantly larger in the CITShift compared to the CITMem, *t*(72) = 2.80, *p* < 0.007.

#### Detection efficiency

Measurement of response latency differences across experimental conditions can lead “to an increased likelihood of finding spurious overadditive interactions” (Faust et al., 1999, p. 777), which could determine an artificial inflation of effect size. The authors recommended *z*-score transformations to augment traditional analyses of raw response latencies. Also, Bush et al. (1993) recommended the use of the *z*-score to remove the influence of individual differences in overall mean response latency within a single group. To eliminate individual differences in responsivity, within-question standardized scores were computed by subtracting the mean of all five responses (one probe and four irrelevants) from the response to the probe and dividing that by the standard deviation of all five values (Ben Shakhar, 1985; Meijer et al., 2007). These standardized scores were then averaged over questions in order to produce a single detection score for the CIT, CITMem, and CITShift (Meijer et al., 2007).

According to signal detection theory, the efficiency of detection may be assessed by considering the degree of separation between the distributions of the detection measure for the innocent and the guilty conditions. Although we included only guilty participants in our study, the distribution of the detection score for innocent individuals can be estimated (Carmel et al., 2003; see also Meijer et al., 2007). Our signal detection parameters were based on a comparison with a simulated innocent group consisting of 73 participants. Following the procedure proposed by Carmel et al. (2003), we generated an innocent group by drawing five values randomly from a standard normal distribution. One value (as the “probe”) was standardized relative to the mean and standard deviation of all five values. The computation was repeated five times and the new values were averaged to obtain a score for one innocent participant (Meijer et al., 2007).

We also analyzed the possibility of increasing detection efficiency by combining measures of concealed information. Using the method described by Nahari and Ben-Shakhar (2011) and by Hu and Rosenfeld (2012), we averaged the *z scores* from CIT and the *z scores* from CIT Shift into a new combined measure.

After we computed the distance (in standard deviation units) between the centers of the two distributions (*d*′), we derived the *area under the receiver operating characteristic* – ROC (Ben Shakhar and Elaad, 2003). The *area under the ROC curve (AUC)* represents the degree of separation between the distributions of the response time from guilty and innocent participants. It varies between 0 and 1 (perfect detection level), with a chance level of 0.5 (Hu and Rosenfeld, 2012). The *d*′ and the *AUC* for each condition are displayed in Table 1.

**Table 1:** **Means, Standard deviations, standardized differences (*d*′) and area under the curve (AUC) for CIT, CITMem, CITShift, and the combination of CIT and CITShift for the Guilty and Innocent Conditions**.

Measure	Mean *z* guilty	Standard deviation guilty	Mean *z* innocent	Standard deviation innocent	*d*′	AUC
CIT	0.48	0.37	−0.04	0.31	1.54	0.86
CITMem	0.53	0.43	−0.03	0.38	1.39	0.83
CITShift	0.59	0.26	−0.02	0.43	1.71	0.88
CIT and CITShift	0.53	0.32	−0.08	0.37	1.75	0.89

*Table 1 reveals that *d*′ values for the CIT, CITMem, and CITShift were 1.54, 1.39, and 1.71, respectively. The *d*′ value for the combination of CIT and CITShift was 1.75. The areas under the ROC curve (*AUC*) were 0.86 for the CIT, 0.83 for the CITMem, 0.88 for the CITShift, and 0.89 for the combination between CIT and CITShift (all other combinations had equal or lower AUCs compared to individual measures)*.

#### Intraindividual bootstrap analysis

To allow for a more in-depth testing of probe versus irrelevant differences within an individual, data from each condition were bootstrapped (Wasserman and Bockenholt, 1989) and hit rates were subsequently calculated. After excluding incorrect behavioral responses and artifacts, a computer program draws, with replacement, a set of individual probe reaction times equal to the number of accepted probe trials in each block and also draws (with replacement) an equal number of irrelevant reaction times, selected randomly from the irrelevant trials. Next, a difference score is obtained by subtracting the mean irrelevant reaction times from the mean probe reaction times. This process is repeated 500 times (Verschuere et al., 2009), resulting in a distribution of 500 differences scores. If the mean difference score minus 1.29 times the standard deviation is greater than zero, it can be concluded with 90% confidence that the probe reaction times are slower than the irrelevant ones.

Bootstrapping of the CIT reaction times resulted in a hit rate of 67%, i.e., for 49 out of 73 participants concealed information was detectable through their slower responses on probe stimuli. For the CITMem, a hit rate of 64% was computed, while for CITShift, 68% of the participants displayed a reaction time for probes that sufficiently deviated from that for irrelevant stimuli to be of diagnostic value.

### Response accuracy

#### CIT accuracy

Additional analyses regarding performance accuracy according to stimulus type were conducted, in order to ensure the comparability of the current procedure with previous data reported by studies using similar methodology (e.g., Seymour et al., 2000). First, mean percent correct for responses to irrelevants and for deceptive responses to probes were calculated (see Figure 1). In order to directly compare percentages for the two stimulus types, an arcsine transformation was then applied to this percent correct data (Cohen, 1988, cf. Gamer et al., 2007).

First, a two-way repeated-measures ANOVA with Condition (CIT vs. CITMem vs. CITShift) and Stimulus type (probe vs. irrelevant) as within-subject factors was conducted. The results showed that there was a significant effect of Condition, *F*(2, 144) = 31.30, *p* < 0.001, MSE = 0.03, partial η^2^ = 0.30. *Post hoc* pairwise comparisons (with a Bonferroni correction) indicated that subjects were significantly less accurate on both the CIT and the CITShift than on the CITMem (although accuracy on the CIT and on the CITShift did not differ).

There was also a significant main effect of Stimulus type, *F*(1, 72) = 80.86, *p* < 0.001, MSE = 0.01, partial η^2^ = 0.53. Across conditions, accuracy in responses to irrelevants was higher than accuracy in responses to probes, *p* < 0.001 (see Figure 1).

Finally, there was a significant Condition × Stimulus type interaction, *F*(2, 144) = 23.59, *p* < 0.001, MSE = 0.01, partial η^2^ = 0.25. Accuracy in response to irrelevants differed across tasks, *F*(2, 144) = 10.65, *p* < 0.001, MSE = 0.01, partial η^2^ = 0.13, with responses on the CITMem being more accurate than on both CIT and CITShift, *p* < 0.05. Accuracy in response to probes also significantly differed across tasks, *F*(2, 144) = 33.67, *p* < 0.001, MSE = 0.03, partial η^2^ = 0.32. Again, *post hoc* contrasts revealed that accuracy to probes on the CIT and CITShift was significantly lower than accuracy to probes on the CITMem, *p* < 0.05. To investigate the magnitude of the difference between accuracy for irrelevants and probes across conditions, difference scores (accuracy for irrelevant minus accuracy for probes) were calculated for each condition. *Post hoc* paired *t*-tests revealed that the difference between irrelevants and probes was larger on the CITShift, compared to both CIT, *t*(72) = 2.37, *p* < 0.02, and to CITMem, *t*(72) = 6.58, *p* < 0.001, respectively. This difference was also larger in the CIT, compared to the CITMem, *t*(72) = 4.93, *p* < 0.001.

#### Accuracy on the concurrent tasks

A final step was to check for accuracy on the secondary tasks (Mem and Shift). Results showed that accuracy for recalling groups of three on the CITMem was high, mean percent correct = 93.37, SD = 5.62. Comparing memory for probes versus irrelevants (after the arcsine transformation of percent correct data), we found that subjects were significantly more accurate in recalling the last word of the probes, than of the irrelevants, *t*(72) = 7.85, *p* < 0.001.

Overall accuracy in shifting between responses to stimuli written in bold or italics was also high, mean percent correct = 87.24, SD = 11.06. This time, accuracy in shifting responses to probes was lower than accuracy in shifting responses to irrelevants (after the arcsine transformation of percent correct data), *t*(72) = 6.88, *p* < 0.001.

## Discussion

The present study analyzed how introducing an additional executive load impacts the accuracy and efficiency of deceptive responses in the RT-based CIT. We hypothesized that the introduction of a concurrent memory load or of flexible shifting demands along with the primary recognition task would selectively interfere with the executive processes required by deception. Therefore, we expected increased detection accuracy of the RT-based CIT in the two conditions with concurrent executive demands, compared to the traditional CIT. We anticipated that the introduction of more complex shifting demands would affect performance to a larger degree than the memory demands. Finally, we also checked whether performance on the concurrent task was itself affected by CIT stimulus type (probe vs. irrelevant).

The results partially confirmed these predictions, but revealed interesting distinctions between group and individual detection efficiency, and between performance accuracy and response time. First, it should be pointed out that the concealed knowledge effect was confirmed across tasks, with subjects presenting longer RTs and lower accuracy on the probes, compared to the irrelevants. This supports the potential of the two RT-based CIT versions (with additional memory load or set-shifting demands) to distinguish between truthful and deceptive responses.

Looking at group differences between conditions in terms of *RT*, we found that subjects were faster on the CIT than on the versions containing additional memory updating or set-shifting demands. This difference was probably a consequence of the extra time required to deal with the increased cognitive load, which affected preparatory, processing, or execution stages of responses in the dual-task conditions (Pashler, 1994). The result confirms previous findings that have used an interfering WM task in the CIT, which increased RTs to both irrelevants and probes (Ambach et al., 2011). Similar to Ambach’s study, the increase in RTs to probes was larger than the increase in RTs to irrelevants, with the outcome of an increased RT-based detection efficiency in the two conditions that contained interfering tasks, compared to the traditional CIT condition – at least at this group level. Since the design did not allow us to directly contrast the influence of the additional cognitive load on guilty versus innocent participants’ behavior, a next step was to simulate a hypothetical group of innocent subjects.

The comparison between distributions of the guilty group and the simulated innocent group showed that the CIT *d*′ value was slightly below the average effect size (*d*′ = 1.55) computed in the meta-analysis made by Ben Shakhar and Elaad (2003) for the psychophysiological CIT. The ability of all the measures to differentiate between guilty and innocent participants was evident from the *d*′ values. The values of 1.39, 1.54, 1.71, 1.75 for the concealed information measures in this study represent a large effect size (Cohen, 1988). Also, the computed *AUC* showed accurate discrimination for all conditions, with the highest rate for the combined measure (CIT + CITShift). Among the two interfering tasks, the demand to flexibly shift responses on a trial-to-trial basis created the largest discrepancy between responses to probes and to irrelevants, and was also associated with the highest hit rate (68%) among the three conditions, although the differences among them were not significant.

In terms of performance *accuracy*, subjects had fewer errors in a CITMem, compared to the traditional CIT and to the CITShift conditions. Importantly, this effect was visible for all stimulus types, and did not differentiate between them. Increasing demands for attentional control induced by concurrent tasks have not been found to affect simple recognition accuracy (Baddeley et al., 1984; Craik et al., 1996), unless there is a deep encoding of the to-be-recognized items (Hicks and Marsh, 2000). It is plausible that the additional conceptual processing required by the memory task might have led to a deeper encoding and to a better subsequent recognition of the stimuli in the CIT task. Since we could not prioritize one task over another, it could be conjectured that the subjects strategically used a sequential strategy. In this context, not only would the two tasks not disrupt each other, but performance on one task could even be enhanced by a deeper earlier processing of the stimuli within the other. When input stimuli are similar, it is possible for dual-task performance to be enhanced because the “same set of processing machinery could be turned on and used for both” and because overt responses we not incompatible between tasks (Pashler, 1994, p. 221). For instance, when focusing on encoding the last word of the probes, subjects could become more aware of the type of stimulus (probe or irrelevant) for the CIT response. Or conversely, if they first focused on responding to the CIT, recognition of the item as a probe could result in an enhanced memory for the previously encountered stimulus. This was indeed demonstrated by better overall accuracy in probe recall. The prolonged/more intensive processing of the stimuli in this condition was translated into an increase in response time compared to the CIT, leading to a potential speed-accuracy tradeoff that is often found in dual-task contexts (Schumacher et al., 2001).

Why was a similar effect not visible in the CITShift condition? In this case, overall accuracy wasn’t significantly different from the traditional CIT, and further, the crucial difference between truthful and deceptive responses was even enhanced – just as we initially expected for both dual-task conditions. Two types of explanations might account for the different findings resulting in enhanced detection efficiency with this task. Firstly, both lower accuracy on the Shift task and the increased processing time suggest that the concurrent task was more difficult and executive-demanding than the memory load task. This confirms the superior executive demands induced by switching between task sets, when compared to simple memory storage (Oberauer et al., 2003). The conclusion is also supported by the greater reciprocal interference between competing tasks when probes were presented. The result of the interference led to a decrease in performance in both the CIT response (longer RT, lower accuracy compared to irrelevants) and the shifting task (lower accuracy for probes). Secondly, the shifting task targeted the *perceptual* (font type), and not the conceptual dimension of the CIT stimuli. This could have generated a greater incompatibility between the two tasks, affecting the more executive-demanding deception trials more. The literature (Pashler and Christian, 1994) suggests yet another possibility: the two simultaneous overt manual responses elicited by the CITShift interfered to a greater degree than the manual plus vocal – non-simultaneous – response present in the CITMem. Finally, an interesting possibility is that the superior accuracy found in the CITMem could simply be a result of the task providing the participants with regular (self-paced) breaks in order to recall the items. This could help them maintain better focus and diminish the goal neglect induced by the (fast-paced) superimposing of two tasks (such as in the CITShift).

Accuracy on the *concurrent tasks* was high in both conditions, but much higher in the Mem task (93%) compared to both the Shift task (87%) and to the previous investigation of Ambach et al. (2011) using an n-back task (83%). It has been shown that if the memory load is significantly below the subjects’ memory span (in this case, three elements), people’s ability to retain the memory load is usually unaffected by the concurrent task, except for a relative slowing of overall task performance (Pashler, 1994) also noticeable in the present study. Interestingly, probes were better recalled than irrelevant items. This could be a result of previous exposure to the probe stimuli during the mock crime. The preferential recall of probes could also indicate memory enhancement for stimuli with emotional/motivational significance, compared to neutral stimuli (Kensinger and Corkin, 2003). The Shift task revealed an opposite trend, namely poorer shifting accuracy in response to trials containing probes. We have already proposed some explanations suggesting the higher interference between the CIT and Shift tasks in the more executive-demanding tasks containing probes. The results obtained with the concurrent task underline the importance of equating for interfering task difficulty; this could be achieved by a control condition containing only the concurrent tasks, but not targeting items from the CIT.

To summarize, by contrasting general detection efficiency between the three conditions, we found the following. According to the group analyses, both dual-task conditions were superior in discriminating between truthful and deceptive responses. Signal detection parameters based on a comparison with the simulated innocent group showed accurate discrimination for all conditions, but did not reveal the same advantage of the dual-task conditions over the traditional RT-based CIT. This apparent inconsistency is not simply a byproduct of the overall slower responses found in the dual tasks, as revealed by our analyses on standardized data. The most plausible explanation is that some participants in the CITShift condition might have presented extremely large probe-irrelevant differences, which were responsible for the group effect. In the light of the current research, the comparison of differences in response latency at a group level need to be interpreted with caution. Combining analyses performed on raw and transformed data can provide important information regarding the most appropriate interpretation of differences in response latency. The computed hit rates for all conditions were slightly higher than those previously found in other RT-based CIT studies (e.g., hit rate of 56%, Verschuere et al., 2009). However, the hit rates computed in our study were still modest (as compared to 95% discrimination accuracy found by Seymour et al., 2000, although different estimating methods were used in that study). Looking at combinations between CIT versions, a combined measure including both CIT and CITShift showed the highest discrimination efficiency. In terms of accuracy, the demand to flexibly shift between types of responses generated the largest discrepancy between probes and irrelevants, while the additional memory load led to ceiling levels of performance accuracy on the CIT (98% for both probes and irrelevants). Performance accuracy on the concurrent tasks was affected by the type of trial (truthful or deceptive), revealing that these tasks could themselves provide valuable clues for deception detection.

The study extends the existing literature dealing with the impact of interfering tasks on the CIT (Ambach et al., 2008, 2011; Hu et al., 2013) in several ways. In the Ambach and collaborators’ studies the exposure time for each CIT (pictorial) stimulus was very large (10 s) in order to collect physiological measures. The authors themselves state that the longer mean RT to CIT stimuli than in other studies could be responsible for the surprising results of shorter RTs for probes than for irrelevants, possibly suggesting strategic alterations of responses in order to appear innocent. In our study the use of the RT-based CIT, the faster pace of the task (1.2 s per verbal stimulus) led to a greater temporal overlap between the primary and the concurrent task, probably generating a stronger interference. However, the self-paced nature of the task induces a potential confound: individual response speed influences overall task speed because by responding earlier the subject receives the following item earlier. A basic measure of psychomotor speed could be introduced to investigate the impact of this individual difference. A further potential confound might be introduced by using three ISIs. However, an analysis of RTs to each stimulus type separated by the preceding ISI interval (500, 800, or 1100 ms) revealed no significant differences.

Second, in both Ambach et al.’s (2008, 2011), and the Hu et al. (2013) studies the inhibition task was peripheral and did not involve the CIT stimuli themselves. In the present study, the concurrent task involved processing the CIT stimuli themselves (remembering the second word of each item in the CITMem and shifting between CIT stimuli written in bold or italics in the CITShift). Again, this might have increased the interference between the two tasks, and led to differences in accuracy/RT between conditions. Finally, Ambach et al. (2011) consider the assignment of conditions (with or without parallel tasks) to large blocks as a potential limitation of their initial study. They favored rapid switches between conditions in the second study. However, we believe that in our design, this manipulation would have induced additional trial-by-trial switching costs, which would obscure the specific effect of memory load/shifting interference. Thus, a large blocks counterbalanced design was chosen.

In the present study, the detection efficiency (compared to a simulated innocent group) in the conditions in which interfering memory (AUC = 0.83) or shifting (AUC = 0.88) demands were introduced was not significantly larger than in the pure RT-based CIT (AUC = 0.86). This final value is strikingly similar to the one obtained by Hu et al. (2013) for their pure RT-based CIT in the comparison with an authentic innocent group (AUC = 0.86). However, in their case, the introduction of an interfering inhibition task led to a significant improvement in detection efficiency (AUC = 0.94). One possibility is that inhibition plays a more crucial role in the deception processes required by the CIT than memory updating or switching, so that interfering with these inhibitory processes leads to greater disruption of deceptive responses. However, design differences between ours and the Hu et al. (2013) study (e.g., a substantially larger number of trials in our case, the use of a peripheral rather than central interference task in their case, and the use of a simulated versus a real innocent group) makes it problematic to directly contrast the findings of these two studies. Further research is needed to disentangle the differential contributions of inhibition, switching, and memory updating demands to the production and execution of deceptive responses in the CIT, preferably in a unitary interference design such as the one proposed in the present study.

An important limitation of the present study is the fact that the exposure time for each stimulus exceeded 1 s, allowing for potential strategic alterations of response speed (Seymour et al., 2000). This could account for the relatively smaller difference between RTs for irrelevants (50–100 ms) and for probes than those obtained in other studies, in which the difference was approximately 200 ms (Seymour et al., 2000; Seymour and Kerlin, 2008, but see Verschuere et al., 2010, for similar difference values to ours). Other limits include the uneven distribution of the sample by gender, which can affect the generalization of the data from the present investigation.

In accordance with the conclusions made by Meijer et al. (2007), our results also indicate that it is worthwhile to combine several different types of lie detection measures. Future studies should make a direct comparison between the incremental validity of RT-based CIT and an RT-based CIT plus a CITShift. The inclusion of an authentic, rather than a simulated innocent group is also recommended.

Both the RT-based CIT, and the “cognitive load” paradigms are recent developments in deception detection research. They are supported by a growing body of evidence (so far, mostly laboratory) that can inform research into the cognitive mechanisms involved in the deceptive act. The use of an interference design can deepen this understanding, creating a selective disruption of a particular executive skill involved in deception. Theoretically, by experimentally introducing different concurrent tasks, one can speculate with regards to the extent to which a particular executive skill is essential to the deceptive act when disrupted, and thus inform research into the neurocognitive mechanisms involved in deception. An implicit assumption which guides the interpretation of our results is that there is a general mechanism subserving both executive functioning and deceptive responses (Johnson et al., 2004), so that disrupting the efficiency of executive functions would directly impact the way a person constructs and executes the deceptive response. However, there is an open question regarding the possibility to dissociate the executive processes underlying deceptive behavior based on such interference designs. Considering the differences between the two dual-task experimental conditions (different fonts used only in the CITShift, regular breaks provided only by the CITMem), their differential impact on deception detection cannot be directly contrasted. Alternative interference designs which would equate for all these experimental variables and would also separately test for individual proficiency in distinct executive functions could offer valuable insights into the executive mechanisms underlying deceptive behavior.

Could the RT-based CIT plus a concurrent task be potentially implemented in field settings? Our results caution us not to transfer this procedure without further documenting its impact upon both RT and accuracy of responses. In the case of CITMem, while the RT for correct responses (the main output) supports the potential of such interference designs to enhance deception detection, an analysis of response accuracy reveals that there are also more correct responses, which makes their comparison with the CIT questionable in terms of RT. As suggested previously, it is possible that this effect might be a result of the CITMem targeting the very contents of the CIT, leading to an increased/prolonged processing of these contents, and to a better performance in deceptively denying their recognition. Further research should confirm whether the introduction of a CITMem peripheral to the CIT in the rapid-paced version of the CIT might provide an optimal candidate for detecting deception.

The demand to flexibly shift between two types of motor responses in accordance to a perceptual characteristic of the CIT stimuli was found to discriminate best between truthful and deceptive responses (at least at a group level). This result has potential implications for interviewing techniques, especially for those involving visual stimuli. Interviewers can alternate between relevant and irrelevant questions regarding critical stimuli from an investigation. It has been shown that rapid alternations between question types (e.g., relevant and irrelevant/unanticipated questions), differentially affects liars’, and truth-tellers’ responses (Vrij et al., 2009). However, a cautionary note relates to the possibility of using the CITShift as a countermeasure. More specific, deceptive subjects might deliberately focus on the perceptual characteristics of the stimulus and ignore their contents, undermining the deception detection process. An important detail is to use a strict response timing deadline that would not permit the participants to strategically alter their responses (as stressed by Seymour et al., 2000). In addition, the fact that the participant would focus only on the secondary task as a countermeasure and ignore the CIT task (leading to higher error rates) would be reflected in an increased accuracy on this concurrent task and facilitate the detection of deliberate faking.

Finally, our results suggest that in any potential application of the RT-based CIT, participants’ responses should be videotaped and analyzed in terms of response accuracy, consistency, and speed, because the outputs from multiple deception indexes do not necessarily converge.

## Conflict of Interest Statement

The authors declare that the research was conducted in the absence of any commercial or financial relationships that could be construed as a potential conflict of interest.

## Appendix

**Table A1:** **Items (English translation) derived from the mock crime and used in the RT-based CIT**.

Probes	Targets	Irrelevants			
*Psiho MCC**	*Psiho GEN*	Psiho DEZ	Psiho COG	Psiho JUD	Psiho SOC
*Four green*	*Nine brown*	Six yellow	Five orange	One red	Seven black
*Amalia Ciuca*	*Bianca Coman*	Emilia Anton	Marcela Petre	Lavinia Dinu	Simona Matei
*Café Amber*	*Café Dante*	Café Tonka	Café Zebra	Café Antic	Café Bistro
*Bicaz Street*	*Tomis Street*	Tusnad Street	Arges Street	Bacau Street	Galati Street

**The abbreviation *Psiho* XXX stands for the name of the course in Romanian, for example: Psiho GEN, general psychology; MCC, cognitive behavioral modifications; DEZ, developmental psychology; COG, cognitive psychology; JUD, forensic psychology; SOC, social psychology; the students are used with these abbreviations for their courses names*.

## References

[B1] Ambach W. Stark R. Peper M. Vaitl D. (2008). An interfering Go/No-go task does not affect accuracy in a concealed information test. Int. J. Psychophysiol. 68, 6–16 10.1016/j.ijpsycho.2007.11.004 18180065

[B2] Ambach W. Stark R. Vaitl D. (2011). An interfering n-back task facilitates the detection of concealed information with EDA but impedes it with cardiopulmonary physiology. Int. J. Psychophysiol. 80, 217–226 10.1016/j.ijpsycho.2011.03.010 21440579

[B3] Baddeley A. D. Lewis V. Eldridge M. Thomson N. (1984). Attention and retrieval from long term memory. J. Exp. Psychol. Gen. 113, 518–540 10.1037/0096-3445.113.4.518

[B4] Ben Shakhar G. (1985). Standardization within individuals: a simple method to neutralize individual differences in skin conductance. Psychophysiology 22, 292–299 10.1111/j.1469-8986.1985.tb01603.x 4011799

[B5] Ben Shakhar G. Elaad E. (2003). The validity of psychophysiological detection of information with the guilty knowledge test: a metaanalytic review. J. Appl. Psychol. 88, 131–151 10.1037/0021-9010.88.1.131 12675401

[B6] Ben-Shakhar G. (2012). Current research and potential applications of the concealed information test: an overview. Front. Psychol. 3:342 10.3389/fpsyg.2012.00342 23060826 PMC3462434

[B7] Bush L. K. Hess U. Wolford G. (1993). Transformations for within-subject designs: a Monte Carlo investigation. Psychol. Bull. 113, 566–579 10.1037/0033-2909.113.3.566 8316614

[B8] Carmel D. Dayan E. Naveh A. Raveh O. Ben-Shakhar G. (2003). Estimating the validity of the guilty knowledge test from simulated experiments: the external validity of mock crime studies. J. Exp. Psychol. Appl. 9, 261–269 10.1037/1076-898X.9.4.261 14664677

[B9] Christ S. E. Van Essen D. C. Watson J. M. Brubaker L. E. McDermott K. B. (2009). The contributions of prefrontal cortex and executive control to deception: evidence from activation likelihood estimate meta-analyses. Cereb. Cortex 19, 1557–1566 10.1093/cercor/bhn189 18980948 PMC2693617

[B10] Cohen J. (1988). Statistical Power Analysis for the Behavioral Sciences, 2nd Edn Hillsdale, NJ: Lawrence Erlbaum Associates

[B11] Craik F. I. M. Govoni R. Naveh-Benjamin M. Anderson N. D. (1996). The effects of divided attention on encoding and retrieval processes in human memory. J. Exp. Psychol. Gen. 125, 159–180 10.1037/0096-3445.125.2.159 8683192

[B12] Dando C. J. Ormerod T. C. Wilcock R. Milne R. (2011). Change temporal order retrieval: help or hindrance. Cognition 121, 416–421 10.1016/j.cognition.2011.06.015 21861997

[B13] Debey E. Verschuere B. Crombez G. (2012). Lying and executive control: an experimental investigation using ego depletion and goal neglect. Acta Psychol. 140, 133–141 10.1016/j.actpsy.2012.03.004 22627157

[B14] Farwell L. A. Donchin E. (1991). The truth will out: interrogative polygraphy (“lie detection”) with event-related potentials. Psychophysiology 28, 531–547 10.1111/j.1469-8986.1991.tb01990.x 1758929

[B15] Faust M. E. Balota D. A. Spieler D. H. Ferraro F. R. (1999). Individual differences in information processing rate and amount: implications for group differences in response latency. Psychol. Bull. 125, 777–799 10.1037/0033-2909.125.6.777 10589302

[B16] Gamer M. (2011). “Detecting of deception and concealed information using neuroimaging techniques,” in Memory Detection: Theory and Application of the Concealed Information Test, eds Verschuere B. Ben-Shakhar G. Meijer E. H. (Cambridge: Cambridge University Press), 90–113

[B17] Gamer M. Bauermann T. Stoeter P. Vossel G. (2007). Covariations among fMRI, skin conductance and behavioral data during processing of concealed information. Hum. Brain Mapp. 28, 1287–1301 10.1002/hbm.20343 17290371 PMC6871443

[B18] Gombos V. A. (2006). The cognition of deception: the role of executive processes in producing lies. Genet. Soc. Gen. Psychol. Monogr. 132, 197–214 10.3200/MONO.132.3.197-214 17969998

[B19] Hicks J. L. Marsh R. L. (2000). Toward specifying the attentional demands of recognition memory. J. Exp. Psychol. Learn. 26, 1483–1498 10.1037/0278-7393.26.5.1160 11185778

[B20] Hu X. Chen H. Fu G. (2012a). A repeated lie becomes a truth? Front. Psychol. 3:488 10.3389/fpsyg.2012.00488 23162520 PMC3495335

[B21] Hu X. Rosenfeld J. P. Bodenhausen G. V. (2012b). Combatting automatic autobiographical associations: the effect of instruction and training in strategically concealing information in the autobiographical implicit association test. Psychol. Sci. 23, 1079–1085 10.1177/0956797612443834 22894937

[B22] Hu X. Evans A. Wu H. Lee K. Fu G. (2013). An interfering dot-probe task facilitates the detection of mock crime memory in a reaction time (RT)-based concealed information test. Acta Psychol. (Amst.) 142, 278–285 10.1016/j.actpsy.2012.12.006 23376139

[B23] Hu X. Rosenfeld J. P. (2012). Combining the P300-complex trial-based concealed information test and the reaction time-based autobiographical implicit association test in concealed memory detection. Psychophysiology 49, 1090–1100 10.1111/j.1469-8986.2012.01406.x 22681260

[B24] Johnson R. Jr. Barnhardt J. Zhu J. (2004). The contribution of executive processes to deceptive responding. Neuropsychologia 42, 878–901 10.1016/j.neuropsychologia.2003.10.011 14998703

[B25] Kensinger E. A. Corkin S. (2003). Memory enhancement for emotional words: are emotional words more vividly remembered than neutral words? Mem. Cogn. 31, 1169–1180 10.3758/BF03195800 15058678

[B26] Logan G. D. (1979). On the use of a concurrent memory load to measure attention and automaticity. J. Exp. Psychol. Hum. 5, 189–207 10.1037/0096-1523.5.2.189

[B27] Lykken D. T. (1959). The GSR in the detection of guilt. J. Appl. Psychol. 43, 385–388 10.1037/h0046060

[B28] Lykken D. T. (1974). Psychology and the lie detection industry. Am. Psychol. 29, 725–739 10.1037/h0037441 4451301

[B29] Meijer E. H. Smulders F. T. Y. Johnston J. E. Merckelbach H. L. G. J. (2007). Combining skin conductance and forced choice in the detection of deception. Psychophysiology 44, 814–822 10.1111/j.1469-8986.2007.00543.x 17584188

[B30] Miyake A. Friedman N. P. Emerson M. J. Witzki A. H. Howerter A. Wager T. D. (2000). The unity and diversity of executive functions and their contributions to complex “frontal lobe” tasks: a latent variable analysis. Cogn. Psychol. 41, 49–100 10.1006/cogp.1999.0734 10945922

[B31] Morgan C. J. LeSage J. B. Kosslyn S. M. (2009). Types of deception revealed by individual differences in cognitive abilities. Soc. Neurosci. 4, 554–569 10.1080/17470910802299987 18654937

[B32] Nahari G. Ben-Shakhar G. (2011). Psychophysiological and behavioral measures for detecting concealed information: the role of memory for crime details. Psychophysiology 48, 733–744 10.1111/j.1469-8986.2010.01148.x 20958308

[B33] Oberauer K. Süß H.-M. Wilhelm O. Wittmann W. W. (2003). The multiple faces of working memory: storage, processing, supervision, and coordination. Intelligence 31, 167–193 10.1016/S0160-2896(02)00115-0

[B34] Pashler H. (1994). Dual-task interference in simple tasks: data and theory. Psychol. Bull. 116, 220–244 10.1037/0033-2909.116.2.220 7972591

[B35] Pashler H. Christian C. (1994). Bottlenecks in Planning and Producing Vocal, Manual and Foot Responses. Center for Human Information Processing Technical Report.

[B36] Schumacher E. H. Seymour T. L. Glass J. M. Fencsik D. E. Lauber E. J. Kieras D. E. (2001). Virtually perfect time sharing in dual-task performance: uncorking the central cognitive bottleneck. Psychol. Sci. 12, 101–108 10.1111/1467-9280.00318 11340917

[B37] Seymour T. L. Kerlin J. R. (2008). Successful detection of verbal and visual concealed knowledge using an RT-based paradigm. Appl. Cogn. Psychol. 22, 475–490 10.1002/acp.1375

[B38] Seymour T. L. Seifert C. M. Shafto M. G. Mosmann A. L. (2000). Using response time measures to assess “guilty knowledge.” J. Appl. Psychol. 85, 30–37 10.1037/0021-9010.85.1.30 10740954

[B39] Spence S. A. (2004). The deceptive brain. J. R. Soc. Med. 97, 6–9 10.1258/jrsm.97.1.6 14702355 PMC1079256

[B40] Spence S. A. Farrow T. F. D. Herford A. E. Wilkinson I. D. Zheng Y. Woodruff P. W. R. (2001). Behavioural and functional anatomical correlates of deception in humans. Neuroreport 12, 2849–2853 10.1097/00001756-200109170-00019 11588589

[B41] Van Bockstaele B. Verschuere B. Moens T. Suchotzki K. Debey E. Spruyt A. (2012). Learning to lie: effects of practice on the cognitive cost of lying. Front. Psychol. 3:526 10.3389/fpsyg.2012.00526 23226137 PMC3510470

[B42] Verschuere B. Ben-Shakhar G. Meijer E. (2011). Memory Detection: Theory and Application of the Concealed Information Test. Cambridge: Cambridge University Press

[B43] Verschuere B. Crombez G. Degrootte T. Rosseel Y. (2010). Detecting concealed information with reaction times: validity and comparison with the polygraph. Appl. Cogn. Psychol. 24, 991–1002 10.1002/acp.1601

[B44] Verschuere B. Crombez G. Koster E. Van Bockstaele B. De Clercq A. (2007). Startling secrets: startle eye blink modification by concealed crime information. Biol. Psychol. 76, 52–60 10.1016/j.biopsycho.2007.06.001 17656000

[B45] Verschuere B. De Houwer J. (2011). “Detecting concealed information in less than a second: response-latency based measures,” in *Memory Detection*. Theory and Application of the Concealed Information Test, eds Verschuere B. Ben-Shakhar G. Meijer E. (London: Cambridge University Press), 46–62

[B46] Verschuere B. Rosenfeld J. P. Winograd M. Labkovsky E. Wiersema R. (2009). The role of deception in P300 memory detection. Legal Crim. Psychol. 14, 253–262 10.1348/135532508X384184

[B47] Visu-Petra G. Miclea M. Visu-Petra L. (2012). RT-based detection of concealed information in relation to individual differences in executive functioning. Appl. Cogn. Psychol. 26, 342–351 10.1002/acp.1827

[B48] Vrij A. Fisher R. Mann S. Leal S. (2006). Detecting deception by manipulating cognitive load. Trends Cogn. Sci. (Regul. Ed.) 10, 141–142 10.1016/j.tics.2006.02.003 16516533

[B49] Vrij A. Granhag P. A. Mann S. Leal S. (2011). Outsmarting the liars: toward a cognitive lie detection approach. Curr. Dir. Psychol. Sci. 20, 28–32 10.1177/0963721410391245

[B50] Vrij A. Leal S. Granhag P. A. Mann S. Fisher R. P. Hillman J. (2009). Outsmarting the liars: the benefit of asking unanticipated questions. Law Hum. Behav. 33, 159–166 10.1007/s10979-008-9143-y 18523881

[B51] Vrij A. Mann S. Fisher R. Leal S. Milne B. Bull R. (2008). Increasing cognitive load to facilitate lie detection: the benefit of recalling an event in reverse order. Law Hum. Behav 32, 253–265 10.1007/s10979-007-9103-y 17694424

[B52] Walczyk J. J. Roper K. S. Seemann E. Humphrey A. M. (2003). Cognitive mechanisms underlying lying to questions: response time as a cue to deception. Appl. Cogn. Psychol. 17, 755–774 10.1002/acp.914

[B53] Wasserman S. Bockenholt U. (1989). Bootstrapping: applications to psychophysiology. Psychophysiology 26, 208–221 10.1111/j.1469-8986.1989.tb03159.x 2727223

